# Hard Gelatin Capsules with Alginate-Hypromellose Microparticles as a Multicompartment Drug Delivery System for Sustained Posaconazole Release

**DOI:** 10.3390/ijms25137116

**Published:** 2024-06-28

**Authors:** Katarzyna Kruk, Katarzyna Winnicka

**Affiliations:** Department of Pharmaceutical Technology, Medical University of Białystok, Mickiewicza 2C, 15-222 Białystok, Poland; katarzyna.kruk@umb.edu.pl

**Keywords:** microparticles, alginate, hypromellose, spray drying, multicompartment drug delivery systems, posaconazole, hard gelatin capsules

## Abstract

Microparticles as a multicompartment drug delivery system are beneficial for poorly soluble drugs. Mucoadhesive polymers applied in microparticle technology prolong the contact of the drug with the mucosa surface enhancing drug bioavailability and extending drug activity. Sodium alginate (ALG) and hydroxypropyl methylcellulose (hypromellose, HPMC) are polymers of a natural or semi-synthetic origin, respectively. They are characterized by mucoadhesive properties and are applied in microparticle technology. Spray drying is a technology employed in microparticle preparation, consisting of the atomization of liquid in a stream of gas. In this study, the pharmaceutical properties of spray-dried ALG/HPMC microparticles with posaconazole were compared with the properties of physical mixtures of powders with equal qualitative and quantitative compositions. Posaconazole (POS) as a relatively novel antifungal was utilized as a model poorly water-soluble drug, and hard gelatin capsules were applied as a reservoir for designed formulations. A release study in 0.1 M HCl showed significantly prolonged POS release from microparticles compared to a mixture of powders. Such a relationship was not followed in simulated vaginal fluid (SVF). Microparticles were also characterized by stronger mucoadhesive properties, an increased swelling ratio, and prolonged residence time compared to physical mixtures of powders. The obtained results indicated that the pharmaceutical properties of hard gelatin capsules filled with microparticles were significantly different from hard gelatin capsules with mixtures of powders.

## 1. Introduction

Microparticles are multicompartment drug formulations, characterized by a highly developed surface area and a diameter of usually 1–500 µm [1]. Microparticles consist of active substances in a molecular dispersion in a matrix formed from natural or synthetic polymers. The purpose of developing multicompartment drug delivery systems is to improve the solubility of poorly water-soluble drugs and, as a result, to increase their bioavailability and to reduce side effects. Microparticles might also be characterized by controlled or prolonged drug release [1,2]. One of the technological methods employed to prepare microparticles is spray drying. This technique involves the atomization of a liquid form, containing a drug substance dissolved, suspended, or emulsified in a polymer solution, in a stream of gas. The spray-drying process consists of three steps, spraying the liquid through a nozzle, evaporating the solvent through a stream of gas, and collecting the resulting product in a receiver. Droplets of small size and highly developed surface area are obtained, during rapid solvent evaporation (seconds, milliseconds), which renders the process safe for substances sensitive to the temperature. Spray drying is characterized by a number of advantages, such as the reproducibility of the process, low cost, simplicity, and obtaining particles with a spherical shape that affects powder flowability, important in the context of filling hard capsules [3,4]. Modulation of the drying process parameters enables an effect on the efficiency of the process and the quality of the obtained microparticles, such as the size or moisture content.

Capsules are a common and frequently encountered solid drug form. Capsules might provide a useful reservoir for multicompartment drug forms, such as microparticles and pellets. The polymer most commonly applied to prepare hard capsules is gelatin, which dissolves in hot water and in the environment of gastric fluid. Compared to tablets, the production of capsules entails the lower utilization of excipients and does not require the application of a compression force, which might destroy the delicate structure of certain materials, such as microparticles [5,6].

Sodium alginate (ALG) is a natural polymer with a polysaccharide structure and anionic character, most often obtained from algae of the *Phaeophyta* family. The mannuronic and guluronic acid residues present in the structure of the ALG chain are linked by β-1,4-glycosidic bonds. The polymer is characterized by numerous advantages, which include high water solubility and ease of gel formation, inert character, good mucoadhesive properties, biocompatibility, and non-toxicity. Producing ALG-based microparticles using spray drying leads to satisfactory results, considering the process yield, encapsulation efficiency, and drug loading [7]. On the other hand, ALG possesses certain limitations, which include precipitation at a low pH (<3), which can limit the release of the active ingredient, and low mechanical strength. Due to these constraints, ALG is combined with other polymers, e.g., cellulose derivatives [7,8,9,10].

Hydroxypropyl methylcellulose (hypromellose, HPMC) is the most widely employed cellulose derivative in the pharmaceutical industry [11]. It belongs to the hydrophilic polymers, a semi-synthetic cellulose derivative with an etheric structure and good solubility in water, with the formation of a viscous colloidal solution. Both, HPMC and ALG are classified as generally recognized as safe (GRAS) by the Food and Drugs Administration (FDA) [12]. HPMC appears under various trade names in a number of varieties with different viscosities. It is characterized by gelling ability and excellent mucoadhesive properties. Due to the ability to swell and gel formation, it is applicable in the extended-release and controlled-release drug forms, as well as in mucoadhesive formulations. Given the lack of electrical charge, HPMC is insensitive to changes in pH in the gastrointestinal tract and exhibits reduced risk of interactions with other excipients [11,13,14,15].

Posaconazole (POS) is a relatively new substance with antifungal activity, belonging to the azole group. The POS mechanism of action involves the inhibition of ergosterol synthesis, which is necessary for fungal cell wall construction. POS exhibits antifungal activity against a remarkably broad spectrum of pathogens, which include *Aspergillus* spp. and *Fusarium* spp., and is approved by the FDA for oral use [16,17]. POS is also effective against *Candida* spp. (a very common cause of vaginal fungal infections) and against species resistant to other azoles (fluconazole or itraconazole). Topical drug administration for vaginal candidiasis treatment is advisable, since it was shown that it is as effective as oral therapy. Moreover, it was demonstrated, in clinical trials, that POS is effective in treating of mucosal candidiasis [18,19]. Therefore, designed formulations were tested in both gastric and vaginal conditions. POS belongs to Class II according to the Biopharmaceutics Classification System (BCS) [20], and its absorption is limited by poor solubility in physiological fluids. Its bioavailability is also highly variable depending on the concomitant intake of food, gut motility, and gastric acidity [21]. Multicompartment mucoadhesive drug formulation–microparticles obtained through a relatively new method of spray drying provide a high-drug-release surface and sustained-drug-release profile [22]. The aim of this study was to compare the pharmaceutical properties of ALG-HPMC microparticles containing POS obtained via spray drying with powder mixtures with the same composition for oral and vaginal administration. Tested formulations were encapsulated in hard gelatin capsules as a practical reservoir for a single dose allowing for simple oral or vaginal administration.

## 2. Results and Discussion

### 2.1. Formulation Characteristics

The formulation composition was established based on our previous study [23] referring to the impact of various ratios of HPMC on the pharmaceutical properties of ALG microparticles. Microparticles with polymer ratios of 1:3 and 1:5 were characterized by the most favorable drug release in 0.1 M HCl and SVF, respectively. They were characterized by significant mucoadhesive and swelling properties compared to other compositions and possessed antifungal activity against *Candida albicans*, *Candida parapsilosis*, and *Candida krusei*; therefore, they were selected for this study. Microparticle formulations MP1 and MP2 were obtained via the spray-drying technique, and powder mixtures Mix1 and Mix2 were blended in a mortar. Microparticles and mixtures of powders were placed in hard gelatin capsules as a functional drug container to obtain a simple dosage preparation. 

Powder flow is an important parameter affecting tablet manufacturing and hard capsule filling processes [24]. The production of pharmaceutical solid drug forms involves several procedures, including flow through hoppers, sieving, mixing, and filling. The efficiency of these processes depends on the powder properties, such as fluidity and bulk density, which affect the quality of the final product [25]. Powder flowability might also be affected by the manufacturing process (spray drying, grinding, crystallization) [26]. Good powder flow properties facilitate accurate filling of the tablet machine matrix and the hard capsule and affect the uniformity and reproducibility of the process [27]. The powder flow is influenced by many physical parameters, which include the powder particle size, particle size distribution, particle surface roughness, and moisture content [28]. As the particle size decreases, the powder flow deteriorates, as van der Waals interactions between powder particles begin to dominate over gravitational forces; therefore, particles are prone to agglomeration [25].

The repose angle is one of the basic parameters of measuring powder flow. It involves pouring a powder mass placed in a funnel through a hole onto a circular plate. The spilled powder forms a cone of a certain height, which is employed to evaluate the angle of repose utilizing the tg α calculation. The higher the internal friction of the powder particles, the higher the cone formed and the higher the calculated angle of repose. Thus, better powder flow is recorded for lower cone heights and smaller values of angle of repose [29,30]. All formulations, aside from Mix1, were characterized by less than a 30° angle of repose value (Table 1); thus, they were characterized by good or very good flow. 

The bulk and tapped density of powders are other parameters applied to evaluate the flowability of powders. They determine the ratio of the mass of the powder to the volume occupied by the loose powder (bulk density) and after a certain number of taps (tapped density). Bulk and tapped density values serve to calculate Carr’s index (CI) and Hausner’s ratio (HR). Based on pharmacopeial criteria, the values of these parameters enable a powder flowability assessment. Powders characterized by low values of CI and HR exhibit better flow than those with higher values of these parameters [31]. CI and HR are employed to evaluate the flow of powders since their values calculated from powder density measurements are related to internal friction. Low internal friction indicates that the powder cannot withstand its own gravitational force; thus, it becomes compacted when the bulk density is measured. Further compaction induced by taping does not lead to a significant volume reduction, and the powder displays a low CI or low HR. The opposite is true for powders with high internal friction, which counteract the gravitational force of the powder mass during the bulk density test. The following compacting of the powder overcomes the internal friction via the impact force, and the powder significantly reduces its volume; hence, higher CI and HR values are obtained in the test. Therefore, powders are estimated to have poor flowability when they undergo high compression during compaction. Based on the results received, microparticles obtained via the spray drying were characterized by lower CI and HR values than physical powder mixtures; however, the flowability of MP1 and MP2 formulations according to pharmacopeial criteria was described as poor, while for Mix1 and Mix2 powder formulations, it was very poor (Table 1). 

Moisture content is a key parameter with regard to the stability of hard capsules. Gelatin, the polymer most commonly used in hard capsule technology, is relatively sensitive to changes in the environmental moisture content [32]. The results of the test are presented in the Table 1. Microparticles possessed a slightly lower moisture content than powder mixtures. The capsules were prepared using a Capsunorm^®^ capsule machine. An evaluation of the content uniformity of the capsules revealed no significant variations from the declared content (50 mg).

### 2.2. In Vitro Release Study

Drug solubility is crucial in terms of their absorption and bioavailability. It is also essential in order to establish sink conditions and to design an in vitro drug release test. As POS is administrated orally for prophylaxis and the treatment of invasive systemic mycosis and vaginally for fungal infections of the reproductive tract, the release study was performed in two acceptor fluids, 0.1 M HCL or SVF, imitating the environment of the stomach and vagina, respectively. POS demonstrated very low solubility in water and SVF, at 0.77 ± 0.14 µg/mL and 0.45 ± 0.13 µg/mL, respectively (Table 2). Since POS is chemically described as a weak alkali, its solubility in 0.1 M HCl is higher (954.68 ± 112.41 µg/mL). POS solubility in SVF increased significantly after adding 1% surfactant (sodium dodecyl sulfate, SDS), from 0.45 ± 0.13 µg/mL to 1349.12 ± 150.50 µg/mL. Microparticles and mixtures of powders were characterized by increased solubility in each environment compared to pure POS; however, there was no significant improvement in solubility in 0.1 M HCl when comparing microparticles to mixtures of powders. The POS solubility in water and SVF for microparticles MP1 and MP2 was only slightly higher than that for physical mixtures of powders Mix1 and Mix2.

**Figure 1:**
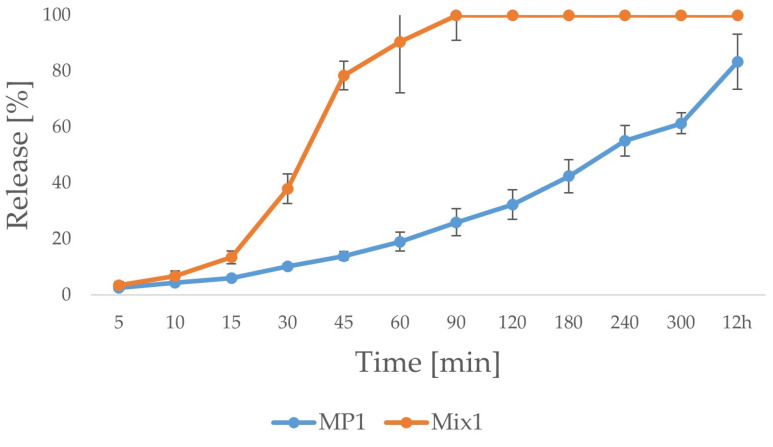
In vitro POS release from capsules containing MP1 and Mix1 formulations in 0.1 M HCl (*n* = 3).

According to our previous paper concerning the pharmaceutical properties of ALG and ALG-HPMC microparticles containing POS with different proportions of both polymers [23], two formulations were selected for this study. Considering the pharmaceutical properties of previously designed ALG-HPMC microparticles, those with ALG to HPMC ratios 1:3 and 1:5 were chosen as oral and vaginal drug-delivery systems for POS, respectively. Microparticles with a polymer ratio of 1:3 possessed the most favorable drug-release profile in 0.1 M HCl, and microparticles with a polymer proportion of 1:5 were characterized by the most beneficial POS release profile in SVF. Selected formulations were also characterized by strong mucoadhesive properties. The microparticle matrix was prepared from a mixture of ALG and HPMC, considering the properties of pure ALG that significantly limit the drug release in an acidic environment. At pH below 3, ALG precipitates and forms a shell, which reduces the diffusion of the liquid inside the drug formulation and consequently limits drug release. The enrichment of the formulation with HPMC, a hydrophilic polymer, easily soluble in 0.1 M HCl, enabled an increase in the solubility of the drug form in gastric conditions and reduced the release time by up to few hours. The differences observed in the release profile for microparticles and mixtures of powders were also due to the different polymer contents and the method of sample preparation. The release study in 0.1 M HCl medium was conducted over 12 h (Figure 1). Despite the fact that there were no significant differences between microparticles MP1 and mixture of powder Mix1 in solubility in 0.1 M HCl, significantly faster release was observed for capsule formulation Mix1 containing a physical mixture of powders compared to microparticles (MP1). Approximately 80% of the released POS was recorded after 45 min for Mix1, while for MP1, it was after 12 h. In addition, during the process of drug release from the Mix1, a rapid increase in the drug concentration in the acceptor fluid was observed between 15 and 45 min of the test (from 13.48 ± 2.26% to 78.41 ± 5.04%). The release from the MP1 formulation was more even and gradual, and no rapid drug release was recorded (10.19% ± 0.87% after 30 min, 19.01 ± 3.39% after one hour, 42.40 ± 5.90% after 3 h). The Mix1 formulation released 100% of the drug in just 1.5 h, while the MP1 released about 83% of the drug in 12 h. This tendency might be related to the microparticle structure and interactions between polymers and POS created during the spray drying. The drug is evenly dispersed and enclosed within the polymer matrix. In contact with 0.1 M HCl, the matrix did not dissolve rapidly; thus, it swelled and formed a gel limiting the drug release. There were no such impacts for the physical mixture of powders.

**Figure 2:**
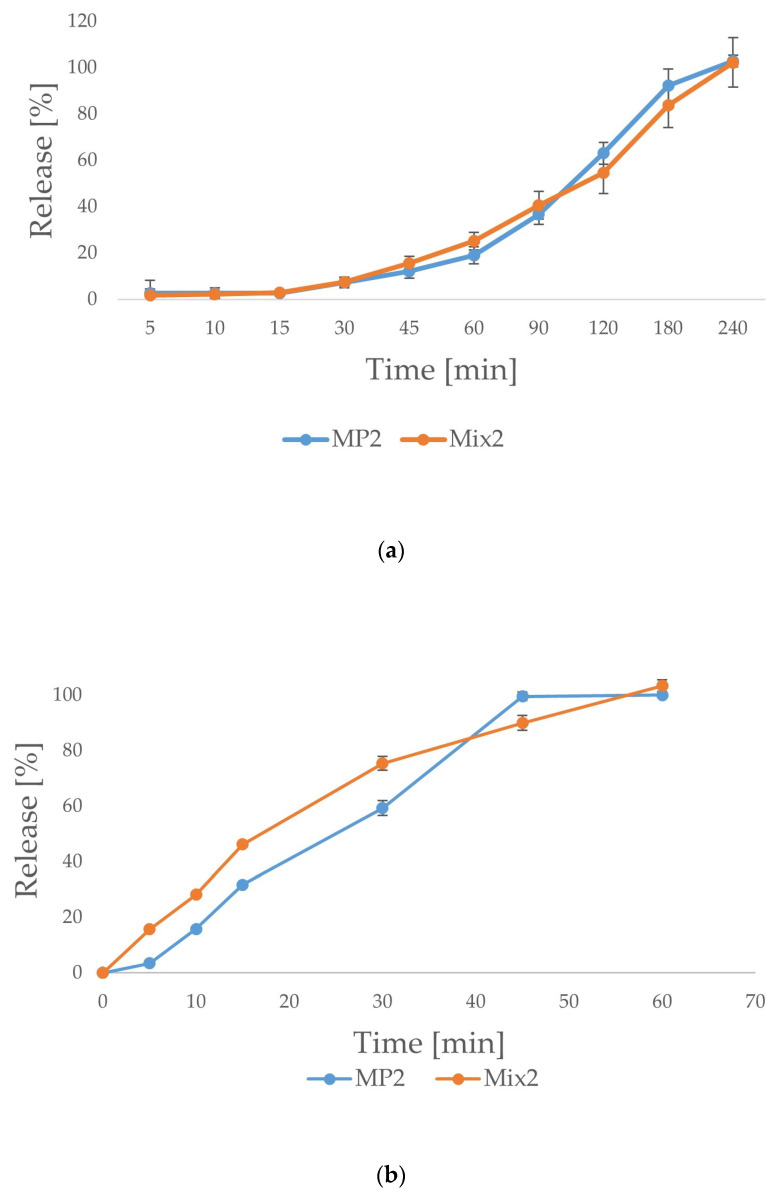
In vitro POS release from MP2 and Mix2 formulations in SVF (n = 3). (**a**)—capsules, (**b**)—after spilling out of the capsules into the release baskets.

The results of the release study for the MP2 and Mix2 formulations carried out in SVF indicated that there were no significant differences in the release process of the drug from gelatin capsules containing the MP2 and Mix2 formulations, despite the fact that solubility in SVF with surfactant was higher for microparticles MP2 than the mixture of powders Mix2. The release profiles were similar with only minor differences (in the time range between 45 min to 1.5 h, the amount of POS released was higher by a few percentage points for the Mix2 formulation, while at time points of 2 h and 3 h, the MP2 formulation possessed a higher release rate by about 10 percentage points) (Figure 2a). Finally, complete drug release was recorded for both formulations after 4 h of testing. An additional variable affecting the POS release profile in this study was the use of hard gelatin capsules. A faster disintegration of the capsules in 0.1 M HCl compared to SVF medium was observed. Despite the good solubility of the ALG-HPMC complex in SVF, the drug release process might be impaired by the presence of the gelatin capsule. During the study, gelatin capsule residues were present for considerably extended periods of time compared to the test in hydrochloric acid, in which the capsules degraded more rapidly. In order to overcome the influence of the gelatin capsule on the release profile, the assay was also conducted for MP2 and Mix2 formulations after spilling out of the capsules into the baskets (Figure 2b). In comparison to the previous test, faster POS release was observed. Approximately 16% of the drug was released from the MP2 formulation after 10 min and from the Mix2 formulation after 5 min. About 100% of POS was released after 45 min from the MP2 formulation, when the complete drug release from the Mix2 formulation was observed after 1 h. Over the course of the test, a greater amount of POS released was noted from the Mix2 formulation than from MP2 for up to 30 min of the assay. Microparticles (MP2) wetted faster and more uniformly than the powder mixture (Mix2). At the moment of complete wetting of microparticles (MP2), in the mass of the powder mixture (Mix2), an unwetted core was present. As a result, the MP2 formulation dissolved more rapidly than Mix2, which was reflected by the faster POS release. The difference in POS release profiles between microparticles and powder mixtures observed in acidic medium were more prominent than in SVF. This might be related to the different solubilities of polymers, since ALG is characterized by good solubility in SVF and poor solubility in 0.1 M HCl. HPMC is soluble in both environments. POS release from MP1 was slower than from Mix1 due to the drug enclosed in the structure of microparticles and the retarded dissolution of the drug form because of the presence of ALG, which precipitates in pH below 3 and limits POS release. In the Mix1 formulation, HPMC dissolved readily in acid and enabled the release of POS, which was only simply mixed with polymers. The results of the release assay in SVF showed only slight differences between microparticles and powder mixtures, as both polymers are characterized by good solubility in SVF. However, formulations MP2 and Mix2 showed more extended POS release when placed in hard gelatin capsules.

The received results were applied using certain mathematical models as zero-order and first-order kinetics, the Higuchi model, the Hixson–Crowell model, and the Korsmeyer–Peppas model to estimate the kinetics of POS release (Table 3). The mathematical modeling demonstrated that the release kinetics were dependent on the medium applied, formulation composition, and method of manufacturing. The highest regression correlation coefficient for formulations MP1 and Mix1 were observed in the Hixson–Crowell model, which also indicated erosion and diffusion as release mechanisms. High R^2^ values for the MP1 formulation were also observed in the zero-order kinetics and Korsmeyer–Peppas models. In the zero-order kinetics model, the R^2^ value was significantly higher for MP1 than the Mix1 formulation, which indicated more gradual and concentration-independent POS release from the MP1 formulation. POS release observed for formulations MP2 and Mix2 was concentration-independent, since it was characterized by zero-order kinetics, due to high values of R^2^ in this mathematical model. The lowest values of the regression correlation coefficient were registered in the first-order kinetics model for all four formulations. After spilling out of gelatin capsules, the MP2 and Mix2 formulation POS release kinetics changed. The highest regression correlation coefficient for the MP2 formulation was observed in zero-order kinetics and the lowest was in first-order kinetics. POS was released from the Mix2 formulation after spilling out in accordance with Higuchi or Hixson–Crowell models, since regression correlation coefficients were at 0.99. The diffusion exponent (*n*) in the Korsmeyer–Peppas model ranged from 0.79 to 1.47, which confirmed that zero-order kinetics underlied the POS mechanism of release for MP2 formulation. The POS release mechanism for the MP1 formulation was characterized by diffusion and zero-order kinetics [33,34,35].

### 2.3. Disintegration Time Test

A disintegration time apparatus was employed to analyze the capsule disintegration times. The disintegration times ranged from 52.33 ± 4.04 min for Mix1 to 197 ± 12.53 min for MP2 (Table 4). The largest difference was observed between the MP1 and Mix1 formulation. Capsules containing the powder mixture disintegrated about 40 min faster than those containing microparticles. No significant differences were noted between formulations MP2 and Mix2. The disintegration time observed in the study was longer for capsules containing MP2 and Mix2 formulations, conducted in an SVF environment, than the disintegration time of capsules tested in 0.1 M HCl containing MP1 and Mix1 formulations, which may be related to the faster degradation of gelatin in an acidic environment. These observations are consistent with the release study results, when faster release from the MP1 and Mix1 formulations in acidic medium was recorded compared to the release rate from MP2 and Mix2 formulations in SVF, and release from Mix1 was significantly faster than from MP1. The lack of significant differences in the disintegration time in the SVF medium for the MP2 and Mix2 formulations is also cohesive with the release study results in SVF, while no significant differences between formulations were reported.

### 2.4. Residence Time Assay

Residence time on the surface of the gastric or vaginal mucosa was tested for gelatin capsules containing microparticles MP1 and MP2 and mixtures of powders Mix1 and Mix2 (Table 5). Microparticles and mixtures of powders were also assessed after spilling out of the gelatin capsules. Microparticles MP1 and MP2 and powder mixtures Mix1 and Mix2 were placed on the mucosa surface after spilling out of the gelatin capsules and pre-wetting with the suitable medium. Both ALG and HPMC are characterized by mucoadhesive properties, as they possess the ability to form bonds with mucin molecules present in the mucosa. Mucoadhesive drug forms are characterized by extended contact time with the mucosa, resulting in prolonged drug release and improved activity [36,37,38,39]. The retention time of microparticles and mixtures of powders slightly differed in both environments (Table 5). There were no significant differences between the retention times of hard gelatin capsules containing microparticles MP1 and the mixture of powders Mix1 in 0.1 M HCl (23.17 ± 2.71 min and 20.83 ± 3.39 min, accordingly). After spilling out of the capsule and placing it on the gastric mucosa, for microparticles MP1 and mixture of powders Mix1 in 0.1 M HCl, no differences appeared (19.17 ± 4.92 min and 20.83 ± 3.19 min, respectively). The retention time of formulation MP1 increased slightly when the powder substance was loaded in a hard gelatin capsule, while for the Mix1 formulation, no significant differences between the powder form and the capsule containing the formulation Mix1 were noted. The retention time of the capsules containing MP2 and Mix2 and powder forms of MP2 and Mix2 was longer in comparison to MP1 and Mix1 formulations in both forms. The MP2 and Mix2 placed on the vaginal mucosa in an SVF environment after spilling out of the capsules exhibited washout times of 26.67 ± 0.82 min and 35.67 ± 3.72 min, respectively. Interestingly, a significant increase in the retention time to about 90 min was noted for the MP2 formulation after loading in gelatin capsules. During this period, the capsules remained on the surface of the vaginal mucosa and gradually disintegrated. No point of complete capsule detachment from the membrane surface was observed; rather, there was a gradual decrease in the mass of the capsule, in contrast to capsules containing MP1 and Mix1 formulations, which separated completely from the surface of the gastric mucosa. In order to investigate the effect of the gelatin capsule on the retention time in SVF, the assay was also performed for an empty capsule placed on the vaginal mucosa. The study showed an approximate 30 min elution time for an empty hard gelatin capsule. Microparticles placed on the gastric mucosa were not characterized by a longer residence time than the powder mixture. On the other hand, microparticles located on the vaginal mucosa possessed a shorter retention time than the physical mixture of powders. The opposite trend was observed for the same formulations loaded in hard gelatin capsules. A longer retention time was observed for microparticles in both environments, with the greater difference noted in SVF. Interestingly enough, powders mixture (formulations Mix1 and Mix2) placed on the mucosa surface were characterized by unequal wetting by both media. Compared to microparticles, which swelled and soaked evenly throughout the mass, in the powder mixture structure, unwetted nodules were noted during the test, which might disturb POS release.

### 2.5. Mucoadhesion Properties

**Figure 3:**
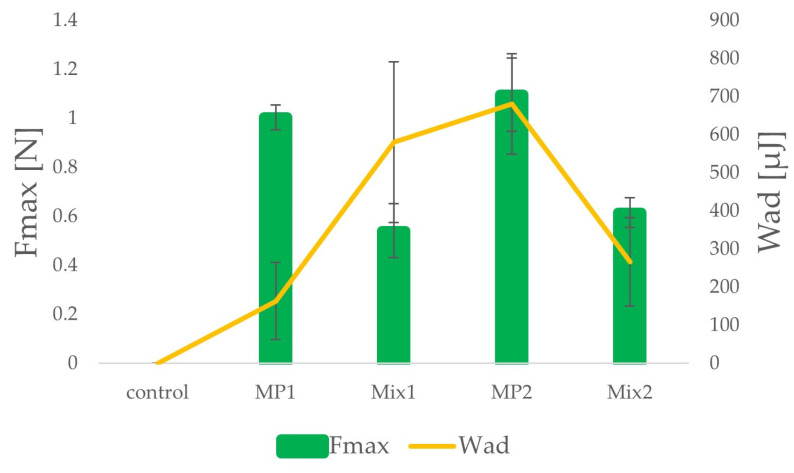
Mucoadhesive properties of formulations MP1, MP2, Mix1, and Mix2 (maximum detachment force F_max_ and work of adhesion W_ad_).

Mucoadhesiveness is described as ability of a material to associate with a mucosa surface through the formation of bonds with mucine. It is beneficial for drug forms containing poorly soluble drugs, since it prolongs the residence time of a formulation on the mucosa surface and consequently enhances drug bioavailability [38,39]. The mucoadhesive properties of the studied formulation were altered and depended on the sample composition and applied medium. The results of the assay are presented in Figure 3. Microparticles obtained via spray drying (MP1 and MP2) possessed higher values of detachment force than (F_max_) powder mixtures (Mix1 and Mix2). A gentle increase in mucoadhesion was observed in SVF for the MP2 formulation compared to the MP1 formulation in 0.1 M HCl (F_max_ 1.10 ± 0.05 N and 1.00 ± 0.15 N, respectively). A similar tendency of an increase in mucoadhesion in SVF compared to in acidic medium was presented by other researchers [18,23]. A significant reduction in the mucoadhesion properties of powder formulations Mix1 and Mix2 was registered in both environments compared to microparticles, since the values of detachment force were at 0.54 ± 0.11 N and 0.62 ± 0.06 N, accordingly. As in the case of microparticles (MP1, MP2), powder mixtures (Mix1, Mix2) were characterized by increased mucoadhesion properties in SVF compared to in an acidic medium (0.1 M HCl), since the values of detachment force (F_max_) were noted at 0.54 ± 0.11 N and 0.62 ± 0.06 N (Mix1 and Mix2 respectively). The stronger mucoadhesion properties observed in the case of microparticles (MP1 and MP2 formulations) compared to simple powder mixtures (Mix1 and Mix2 formulations) might be related to the process of manufacturing. During spray drying, the solvent evaporates in a hot stream of inert gas, and particles of polymers approach, generating bonds and enclosing POS inside the matrix. There was no such phenomenon regarding powder mixtures prepared by grinding in a mortar.

### 2.6. Swelling Ratio

Swelling is the effect of fluid absorption by the polymer. Swelling and forming a gel structure are crucial factors affecting the mucoadhesive properties of microparticles and the drug-release profile, influencing the residence time on the gastric and vaginal mucosa. During microparticle swelling, the polymer chains elongate, revealing the mucoadhesive bonding sites of the polymer, which further contribute to the extended residence time of the drug form on the mucosa surface [40].

**Figure 4:**
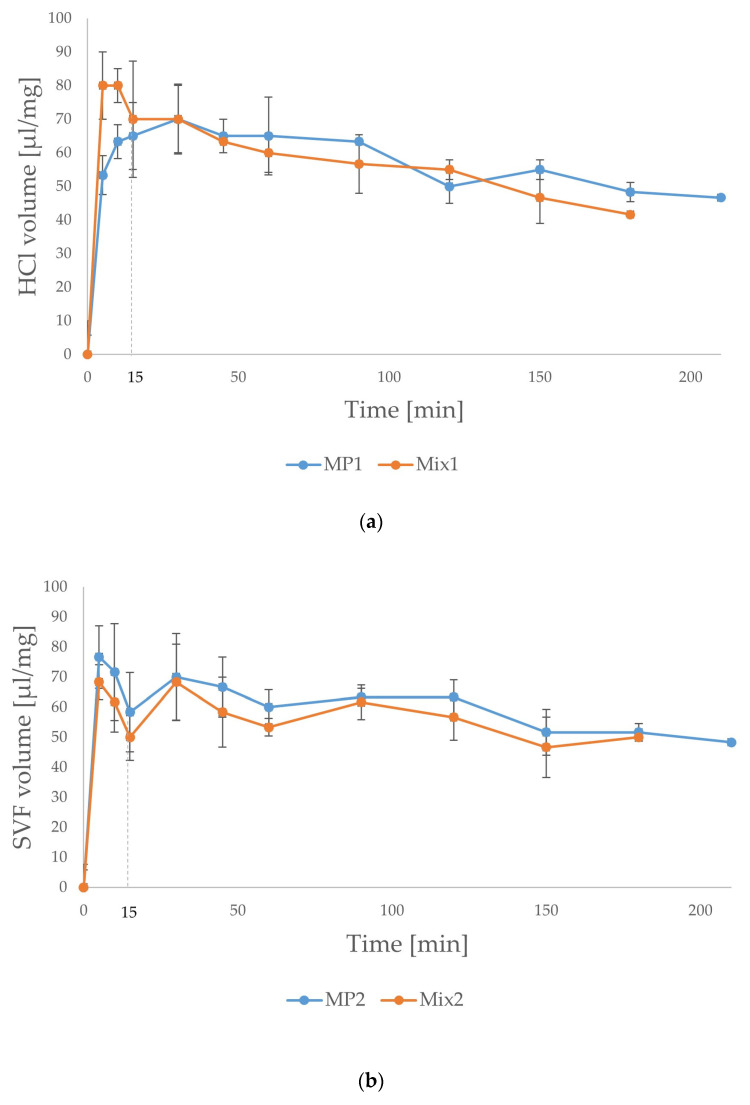
Swelling ratio of microparticles MP1 and MP2 and mixtures of powders Mix1 and Mix2 described as the volume of medium absorbed [µL/mg] (in 0.1 M HCl (**a**) and in SVF (**b**)).

In contact with 0.1 M HCl (pH 1.2) or SVF (pH 4.8), microparticles swelled and the simultaneous erosion of microparticles occurred (Figure 4). However, the swelling rates observed were different in 0.1 M HCl and in SVF. Microparticles MP1 were characterized by an increased swelling ratio after 30 min of the assay. Within the next hour, the swelling plateau established began to gently decrease after 1.5 h. Formulation Mix1, physical mixtures of powders, revealed a different swelling profile. At the two first time points at 5 min and 10 min, higher absorption of the medium was observed compared to microparticles MP1. After 10 min of the assay, swelling of the Mix1 formulation began to gradually decrease. Degradation of the Mix1 formulation in the acceptance medium was observed after 30 min. On the contrary, formulation MP1 did not dissolve during the study. There were no significant differences between microparticles MP2 and the mixture of powders Mix2 during the swelling test in SVF. Formulation MP2 was characterized by a slightly higher swelling ratio compared to Mix2 within the assay time. Both MP2 and Mix2 heavily swelled at the beginning of the test, after 5 min with a decrease in swelling after 15 min. No degradation of SVF was observed during the test, in contrast to the Mix1 formulation in 0.1 M HCl. Formulations MP2 and Mix2 demonstrated higher swelling in SVF than MP1 and Mix1 in 0.1 M HCl, which might be caused by the different solubilities of ALG in HCl and SVF. Since ALG precipitated in pH 1.2, the swelling of microparticles MP1 was slow and gradual, reaching the maximum swelling after 30 min. Precipitated ALG limited medium diffusion into the microparticles and reduced the swelling of HPMC, of which solubility is pH-independent [11]. No such dependence was observed for the Mix1 formulation, since polymers were simply mixed without creating intermolecular bonds. In contact with HCl, HPMC and POS dissolved gradually resulting in degradation of the formulation. In SVF, higher swelling of both formulations was observed, since ALG was negatively charged in a higher pH, which might enhance swelling. The higher medium absorption was due to the polyanionic character of ALG, which facilitated swelling via the steric repulsion of uniquely charged ALG chains [41]. The swelling properties of HPMC are widely known, although they are most often described in the perspective of matrix tablets [42,43]. HPMC is not an ionic polymer, and therefore, the swelling ability might be related to the aggregation of hydrophobic parts of HPMC chains with the formation of transient cross-linking [43]. On the contrary, HPMC swelling was unaffected by the pH of the medium, likewise as in our previous study [23]. 

### 2.7. DSC Analysis

The evaluation of phase transitions and melting points of active substances, polymers, and their mixtures is essential for understanding the relationships between the drug and excipients. Differential scanning calorimetry (DSC) is a method for an appraisal of the thermal properties of drug forms and an evaluation of the association among the polymeric composition, its microstructure, and stability [44,45]. 

**Figure 5:**
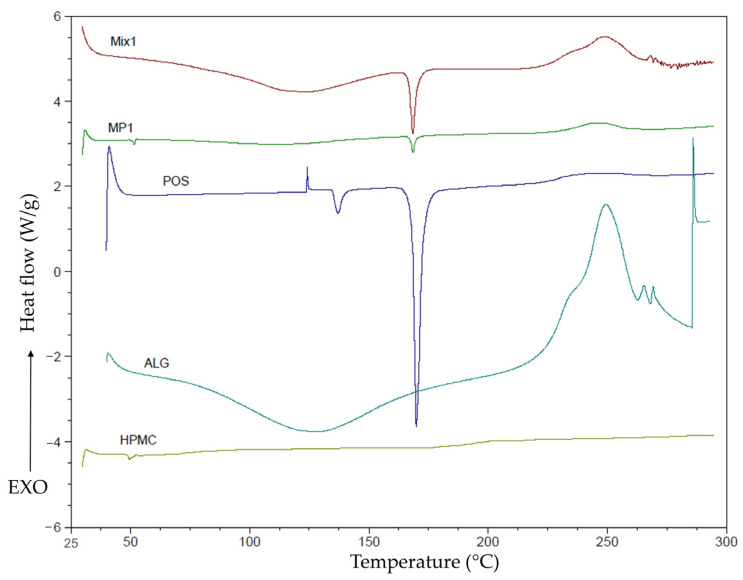
DSC thermogram of ALG, POS, HPMC, MP1, and Mix1 formulations.

DSC analysis was carried out for pure ALG, HPMC, POS, placebo, and MP1 and Mix1 formulations (Figure 5). In the thermogram of pure ALG, a broad endothermic peak was registered between 50 °C and 200 °C, and a sharp exothermic peak was observed at 248 °C. Their incidence might be related to water evaporation with an increasing temperature and the degradation of the polymer structure, respectively. Dudek et al. [46] indicated that the ALG chain might undergo decomposition during the dehydration process, depolymerization, and partial decarboxylation of ALG carboxyl groups resulting in the destruction of saccharide rings. The glass transition temperature of HPMC ranges from 170 °C to 180 °C, and the degradation temperature fluctuates between 200 °C and 250 °C, depending on the HPMC substitution [11]. In the HPMC thermogram, any peaks originating from the melting point were noted, indicating an amorphous state [47]. In the POS thermogram, two sharp endothermic peaks at 138.7 °C and 172.7 °C were recorded, indicating crystalline water loss and the melting point of POS, respectively. 

In the thermogram of the MP1 formulation, a weak peak from the POS melting point at 168.35 °C was observed, and a peak at 248 °C originating from degradation of the ALG structure was recorded. A broad endothermic peak between 50 °C and 200 °C, originating from ALG dehydration, was weakly outlined. In the thermogram of the Mix1 formulation two peaks originating from ALG were observed: a broad endothermic peak in the range of 50–200 °C and a sharper exothermic peak around 250 °C. There was no endothermic peak originating from the loss of POS crystalline water. In the thermogram of the mixture of powders, Mix1 peaks originating from individual components were more pronounced compared to microparticle MP1, suggesting that during spray drying, intermolecular interactions between polymers and POS were presumably created, which might affect the physicochemical properties of the formulation. 

## 3. Materials and Methods

### 3.1. Materials

Sodium alginate (ALG) with medium viscosity (1%, 282 mPa·s, 61% mannuronic acid (M), and 39% guluronic acid (G), and M/G ratio of 1.56) was obtained from Sigma Aldrich (Steinheim, Germany). Hydroxypropyl methylcellulose (HPMC) (Pharmacoat 615, 2%, viscosity 15 mPa·s) was received from Shin-Etsu Chemical Co., Ltd. (Tokyo, Japan). POS was attained from Kerui Biotechnology Co., Ltd. (Xi’an, China). Methanol was procured from Merck (Darmstadt, Germany). Empty hard gelatin capsules, size 00 were obtained from CAPSUGEL-BORNEM (Bornem, Belgium). Water was distilled using a Milli-Q Reagent Water System (Billerica, MA, USA). Simulated vaginal fluid (SVF, pH = 4.2) was prepared by dissolving the following in 1 L of water: 0.018 g bovine albumin, 0.16 g glycerol, 0.222 g calcium hydroxide, 0.4 g urea, 1.0 g acetic acid, 1.40 g potassium hydroxide, 2 g lactic acid, 3.51 g natrium chloride, and 5 g glucose [48]. Nylone membrane filters (0.45 µm) were purchased from Millipore (Billerica, MA, USA). Porcine gastric and vaginal mucosa were received from a veterinary service (Turośń Kościelna, Poland). Excerpts from the porcine mucosa were frozen at −20 °C and stored for a maximum of one month prior to testing. Before the experiment, they were defrosted and cut into smaller parts. This process did not require the agreement of the Local Ethical Committee for Experiments on Animals. All other reagents utilized in the experiments were of analytical grade.

### 3.2. Samples Preparation

Microparticles ALG-HPMC with POS were prepared using spray drying technology utilizing a mini spray dryer B-290 (Büchi, Flavil, Switzerland). The microparticle composition is presented in Table 6. Suitable amounts of polymers were dissolved in water, and an appropriate quantity of POS was homogeneously dispersed in the obtained polymer solutions using a magnetic stirrer (Heidolph Instruments, Schabach, Germany). Prepared dispersions were spray dried under experimentally determined conditions (inlet and outlet temperatures: 150 °C and 86 °C, respectively; aspirator blower capacity, 85%; pressure, 80 mm Hg; and feed rate, 2.1 mL/min). Physical mixtures of powders were prepared by mixing, in a mortar, equal amounts of powders utilized to prepare microparticles.

### 3.3. Angle of Repose

The angle of repose assay was conducted employing the manual powder flow tester (Electrolab EFT-01, Mumbai, India), according to the procedure recommended by the European Pharmacopoeia 11 [49]. An amount of 10 g of each formulation was placed in the funnel of the apparatus. The septum was removed allowing the powder to pour onto a circular platform of a defined diameter of 10 cm. The height of the formed cone served to calculate the angle of repose according to the following formula:

tg α = h/r(1)

where h is the height of the cone, and r is the radius of the platform.

### 3.4. Bulk and Tapped Density

The test was performed in accordance with European Pharmacopoeia 11 guidelines utilizing an Electrolab ETD-1020 Tap Dentity Tester (Electrolab, Mumbai, India). Here, 10 g of each formulation was placed in a measuring cylinder. Density values were calculated based on the read volume covered by the powder without compacting and after 500, 750, and 1250 taps. Carr’s index and Hausner’s coefficient were calculated based on equations according to the European Pharmacopoeia 11 [49]:

CI = (ρ_tap_ − ρ_bulk_)/ρ_tap_ × 100 (2)

HR = ρ_tap_/ρ_bulk_ (3)

where ρ_tap_ is the tapped density, and ρ_bulk_ is the bulk density.

### 3.5. Particle Size

An optical microscope (Motic BA 400, Moticon, Wetzlar, Germany) was employed to evaluate the particles size. All formulations were observed under a 40× magnification, and particles size was measured.

### 3.6. Moisture Content

The moisture content of the prepared formulations was determined using a Radwag WPS 50SX moisture analyzer (Warsaw, Poland). For this purpose, 20 mg of each formulation was placed in an aluminum sheet and heated in the temperature range of 30 °C to 120 °C.

### 3.7. Capsule Preparation

A Capsunorm^®^ hand capsule machine (EPRUS, Bielsko-Biala, Poland) was employed for capsule preparation. The amount of the formulation was adjusted in order to provide 50 mg of POS for each formulation in hard gelatin capsules, size 00.

### 3.8. Evaluation of POS Content in Hard Gelatin Capsules

Twenty randomly selected capsules of each formulation were utilized to test the weight uniformity, and the average weight per capsule and standard deviation were calculated. Ten capsules were picked for the assessment of the drug content uniformity. The capsule contents were quantitatively transferred to flasks containing 20 mL of water and placed overnight in a water bath with a temperature of 25 °C ± 1 °C and a stirring rate of 250 rpm. It was experimentally established that 40 mL of methanol must be added to each flask to dissolve the drug, since POS solubility in water is limited. The solution was filtered using a nylon filter with a pore diameter of 0.45 µm; 0.1 mL of the solution was transferred to a flask, which was filled up to 10 mL with 0.1 M HCl and analyzed spectrophotometrically (Section 3.11).

### 3.9. Solubility Test

The solubility test consisted of placing 3 mL of solvent (water, 0.1 M HCl, SVF, SVF with 1% SDS as surfactant), an excess of pure POS, and tested formulations in 5 mL flasks, which were sealed tightly and vortexed for 1 min. They were transferred to a water bath (36.6 °C ± 0.1 °C) and shaken at 250 rpm for 24 h. After this time, the samples were centrifuged for 10 min at 4000 rpm and filtered using cellulose acetate filters (0.22 µm). Samples prepared in this way were diluted 100-fold with accurate medium and analyzed spectrophotometrically (Section 3.11).

### 3.10. In Vitro Release Profile

According to the procedure described in the European Pharmacopoeia 11, a release testing apparatus (Erweka Dissolution tester type DT 600HH, Heusenstamm, Germany) was applied for the in vitro release profile assessment. Capsules were placed in baskets and immersed in 500 mL of release medium: 0.1 M HCl (pH 1.2) or SVF (pH = 4.2), representing gastric and vaginal conditions, respectively. SVF was enriched with 1% SDS to provide sink conditions. Formulations MP2 and Mix2 were also assessed after spilling out of the capsules into the release baskets in order to precisely register differences in the release profiles. Samples were collected at time points of 5 min, 10 min, 15 min, 30 min, 45 min, 1 h, 1.5 h, 2 h, 3 h, 4 h, 5 h, and 24 h and analyzed spectrophotometrically (Section 3.11).

### 3.11. Spectrophotometric Analysis

The analysis was carried out with a spectrophotometer (Jasco V-750, Tokyo, Japan) at a wavelength of 254 nm. The standard calibration curve was linear over the range of 1–50 g/mL and characterized by correlation coefficients (R^2^) of 0.9968 and 0.9997; the limit of detection (LOD) and limit of quantification (LOQ) were 0.5 g/mL and 1.0 g/mL, (0.1 M HCl) and (SVF), respectively.

### 3.12. Mathematical Modeling of the Release Profile

To evaluate the POS release mechanism, results from the POS release test were assessed using the following mathematical models:

Zero-order kinetics:

F = k × t(4)

First-order kinetics:

lnF = k × t(5)

Higuchi model:

F = kt^1/2^ (6)

Korsmeyer–Peppas model:

F = kt^n^ (7)

Hixson–Crowell model:

1 − (1 − F)^1/3^ = kt (8)

where F is the released drug, k is the constant related to the drug release, and t is the time.

### 3.13. Disintegration Time

A pharmacopeial disintegration time apparatus (Electrolab ED-2L, Mumbai, India) was employed for the assay. The test was conducted at 37 ± 1 °C in 0.1 M HCl (pH 1.2) or SVF (pH = 4.2) for six capsules of each formulation [49]. It consisted of measuring the time for the capsule to disintegrate (until the formulation unit was a soft mass with no non-wetted core).

### 3.14. Residence Time Test

The test was performed with a modified disintegration time apparatus according to Nakamura et al. [50]. The modification consisted of applying of a Plexiglas cylinder moving up and down inside a beaker containing 0.1 M HCl (pH = 1.2) or SVF (pH = 4.2) at 37 ± 1 °C. Fragments of porcine gastric and vaginal mucous membranes of 2 cm^2^ were attached to the inner wall of the beaker above the medium level, onto which tested powder formulations and capsules containing formulations were applied and then immersed in the medium through the pumping motion of the cylinder. The time for complete washing out of the powder and capsulated formulations from the surface of the mucous membranes was measured.

### 3.15. Swelling Ratio

Swelling properties of microparticles containing POS (MP1, MP2) and mixtures of powders with POS (Mix1, Mix2) were assessed with the use of a Franz diffusion cell. Formulations in the amount of 10 mg were placed on the regenerated cellulose membrane (0.45 μm pore size) covering the membrane surface. The receiver chamber was filled up with 0.1 M HCl or SVF thermostated at 37 °C. At the set time intervals (after 5, 10, 15, 30, 45, 60, 90, 120, 150, 180, and 210 min), the receiver chamber was refilled with the medium up to the primary level, which was reduced due to HCl or SVF uptake by the microparticles or mixtures of powders. A graduated microliter syringe (Hamilton, Bonaduz, Switzerland) was applied to refill the medium. The swelling ratio was calculated as a volume of the medium absorbed by one milligram of the formulation [51].

### 3.16. DSC Analysis

Differential scanning calorimetry analysis (DSC) of the unprocessed ALG, HPMC, POS, and spray-dried microparticles was carried using a Mettler Toledo Star TGA/DSC unit. Since the qualitative composition of microparticles and mixtures of powders was the same, MP1 and Mix1 formulations were analyzed in the DSC assay as representative formulations of microparticles and mixtures of powders, respectively. In the DSC analysis, aluminum crucibles with 3–5 mg of weighted samples were heated from 0 °C to 300 °C at 20 °C/min under an argon flow; an empty pan was used as the reference [23].

### 3.17. Mucoadhesive Properties

Mucoadhesive properties were evaluated with the use of a TA.XT Plus Texture Analyzer (Stable Micro Systems, Godalming, UK). Porcine gastric and vaginal mucosa were applied as mucoadhesive layers. After spilling out of gelatin capsules, formulations were moisturized with the respective medium (0.1 M HCl or SVF) and exposed to contact with the mucosa with a 0.5 N force for 120 s. The mucoadhesive properties were assessed by measuring the detachment force (F_max_) and work of mucoadhesion (W_ad_) registered by Texture Exponent 32 Software, version 5.0 [52].

### 3.18. Statistical Analysis

Collected data were evaluated using Statistica 13.3 (StatSoft, Tulsa, OK, USA), utilizing one-way analysis of variance (ANOVA) or a Kruskal–Wallis test. The results achieved were implemented as the mean and standard deviation on the basis of at least three independent experiments.

## 4. Conclusions

The presented results indicate that the pharmaceutical properties of ALG-HPMC microparticles containing POS differ from the powder mixtures of the same qualitative composition formulations. Changes in the release profile of POS were also observed. Drug incorporation into the polymer matrix affected the release profile. In an acidic medium, hard gelatin capsules loaded with microparticles were characterized by prolonged release compared to capsules with a physical powder mixture. Microparticles obtained via spray drying were uniformly wetted by the medium, which might favorably affect the drug release and absorption. Moreover, they were characterized by stronger mucoadhesive properties compared to physical powder mixtures in both environments. Hard gelatin capsules filled with microparticles possessed a longer residence time on the vaginal mucosa surface compared to capsules with a physical powder mixture, what might improve POS bioavailability. The swelling ratio depended on the medium applied, and microparticles and powder mixtures exhibited stronger swelling in SVF than in 0.1 M HCl. Developing hard gelatin capsules filled with ALG-HPMC microparticles with POS via the spray technique led to the acquisition of a multicompartment mucoadhesive drug form with a prolonged release profile in an acidic environment. 

## Figures and Tables

**Table 1:** Characteristics of microparticles MP1 and MP2 and mixtures of powders Mix1 and Mix2 utilized for capsule preparation.

	MP1	MP2	Mix1	Mix2
Drug loading [%]	36.19 ± 1.63	20.59 ± 1.28	Not tested *	Not tested *
Particle size (µm)	14.08 ± 2.05	16.38 ± 2.27	14.93 ± 3.62	14.56 ± 2.15
Moisture content [%]	6.35 ± 0.83	13.67 ± 1.46	7.66 ± 1.52	16.60 ± 2.00
Angle of response [°]	25.01 ± 0.54	18.77 ± 1.03	31.78 ± 1.66	23.10 ± 0.56
Bulk density [g/mL]	0.11 ± 0.00	0.14 ± 0.00	0.38 ± 0.00	0.41 ± 0.00
Tapped density [g/mL]	0.17 ± 0.00	0.21 ± 0.00	0.63 ± 0.01	0.67 ± 0.01
Carr’s index [%]	35.68 ± 0.34	32.85 ± 1.56	39.58 ± 0.94	38.98 ± 1.02
Hausner’s ratio	1.55 ± 0.01	1.49 ± 0.03	1.66 ± 0.03	1.64 ± 0.03

*—substances were blended in precisely defined proportions.

**Table 2:** POS solubility for microparticles MP1 and MP2 and for mixtures of powders Mix1 and Mix2 in comparison to unprocessed POS powder solubility.

Solubility [µg/mL]				
Formulation	Water	0.1 M HCl	SVF	SVF + 1% SDS
POS *	0.77 ± 0.14	954.68 ± 112.41	0.45 ± 0.13	1349.12 ± 150.50
MP1	14.23 ± 1.63	1187.35 ± 36.80	13.92 ± 0.35	1757.90 ± 139.28
MP2	13.02 ± 0.96	1107.39 ± 45.74	12.97 ± 0.25	1755.09 ± 95.23
Mix1	8.51 ± 0.05	1102.13 ± 17.01	8.02 ± 0.21	1340.70 ± 144.83
Mix2	12.42 ± 0.22	1146.63 ± 24.89	12.42 ± 0.18	1794.39 ± 174.03

*—unprocessed POS powder.

**Table 3:** Kinetic modeling of POS release from capsules containing microparticles MP1 and MP2 and mixtures of powders Mix1 and Mix2 (in 0.1 M HCl—A and in SVF—B) and from MP2 and Mix2 formulations after spilling out of the capsules (C).

Formulation	Zero-Order Kinetics		First-Order Kinetics		Highuchi Model		Hixson-Crowell Model		Korsmeyer-Peppas Model		
	R^2^	K	R^2^	K	R^2^	K	R^2^	K	R^2^	K	*n*
A. 0.1 M HCl (pH 1.2)											
MP1	0.99	0.20	0.78	0.009	0.98	3.91	0.99	0.005	0.99	0.18	0.79
Mix1	0.91	1.29	0.78	0.04	0.95	15.49	0.96	0.05	0.98	1.97	1.29
B. SVF (pH 4.2)											
MP2	0.97	0.48	0.83	0.02	0.93	8.34	0.90	0.01	0.94	1.18	1.11
Mix2	0.99	0.46	0.79	0.02	0.95	7.93	0.98	0.01	0.98	1.25	1.19
C. SVF (pH 4.2) after spilling out											
MP2	0.99	2.34	0.79	0.07	0.98	21.12	0.90	0.09	0.96	2.83	1.47
Mix2	0.95	1.58	0.82	0.02	0.99	16.35	0.99	0.06	0.98	0.69	0.77

**Table 4:** Disintegration times of gelatin capsules with microparticles MP1 and MP2 and mixtures of powders Mix1 and Mix2.

Disintegration Time [min]				
	Microparticles		Physical Powders Mixtures	
	MP1	MP2	Mix1	Mix2
0.1 M HCl (pH 1.2)	95.33 ± 1.53	-	52.33 ± 4.04	-
SVF (pH 4.8)	-	197.00 ± 12.53	-	194.67 ± 8.14

**Table 5:** Residence times for hard gelatin capsules containing microparticles MP1 and MP2 and powder mixtures Mix1 and Mix2 and for microparticles MP1 and MP2 and mixtures of powders Mix1 and Mix2 after spilling out of the capsules.

Residence Time [min]				
	Microparticles		Physical Powders Mixtures	
	MP1	MP2	Mix1	Mix2
Capsules	23.17 ± 2.71	89.67 ± 2.52	20.83 ± 3.39	38.33 ± 6.28
After spilling out	19.17 ± 4.92	26.67 ± 0.82	20.83 ± 3.19	35.67 ± 3.72

**Table 6:** Formulation compositions.

	Microparticles		Mixture of Powders	
Formulation	MP1	MP2	Mix1	Mix2
Amount of POS [mg]	50	50	50	50
ALG:HPMC:POS ratio	1:3:1	1:5:1	1:3:1	1:5:1

## Data Availability

Data are contained within the article; raw data are available upon request.
